# The co-construction of a reading assessment measure with adults with Down syndrome: a meaningful literacy approach

**DOI:** 10.3389/fpsyg.2023.1173300

**Published:** 2023-07-20

**Authors:** Pauline Frizelle, Sean O’Donovan, Mary Jolley, Lisa Martin, Nicola Hart

**Affiliations:** ^1^Department of Speech and Hearing Sciences, University College Cork, Cork, Ireland; ^2^Down Syndrome Ireland, Dublin, Ireland

**Keywords:** meaningful reading, adults, Down syndrome, co-construction, assessment, literacy, participatory research

## Abstract

**Introduction:**

The need to develop appropriate measures of broad-based reading-related literacy skills for adults with Down syndrome has been highlighted in the literature. In this study we aimed to co-construct a valid and reliable assessment measure that can be used to document meaningful everyday reading, in adolescents and adults with Down syndrome.

**Methods:**

The study was carried out in two stages. Stage 1 used an inclusive participatory design in which individuals with Down syndrome were research collaborators (*n* = 46). Items to be included in the measure were identified and ecological, face and content validity were established through an iterative process. In stage 2 we examined the reliability of the tool and explored potential relationships between meaningful reading score and (1) age, (2) receptive vocabulary, and (3) reading ability as measured by standardized assessments. In addition, we profiled what a pilot cohort of adults with Down syndrome read (*n* = 33) and how they experience reading in their everyday lives.

**Results:**

Results showed that 46 items were generated for inclusion in the Meaningful Reading Measure (MRM). Our preliminary data showed that the tool has internal and external reliability and ecological and content validity. There were no associations between meaningful reading score and any of the other variables examined. There was considerable variability in items read (range 12–44) which reflected a broad range of reading practices. Adults with Down syndrome identified the importance of reading as a pleasurable activity and as something that aids learning.

**Conclusion:**

The MRM developed here can be used (1) as a reading intervention outcome measure to complement existing standardized tools, (2) to profile meaningful reading in adults with Down syndrome, (3) to guide reading module content, and (4) to capture change in adults’ perceptions of themselves as readers. Future work is needed to establish the tool’s sensitivity to change over time.

## Introduction

Reading skills play an important role in the lives of people with and without Down syndrome. Reading is a key part of human communication; is required to navigate the modern technological world; facilitates participation and inclusion in society; is a recognized goal of human development; and is considered to improve an individual’s overall well-being (Maddox, 2008; Dukes and Ming, 2014; McClure et al., 2015). The value of learning to read has become even more significant in recent years, as how we use written language in society has evolved. A recognition of this change is reflected in the inclusion of literacy as a global target in the UN sustainable development goals. The aim being that “by 2030 all young people and adults across the world should have achieved relevant and recognized proficiency levels in functional literacy that are equivalent to levels achieved at successful completion of basic education” (Global Campaign for Education, 2023). An increasing dependence on technology has resulted in text messaging often replacing conversation, emailing replacing phone calls and search engines replacing reference books and guides. Consequently, reading is key to navigating everyday situations and is required to function effectively both online and in the real world. In the current study, we aimed to co-construct a valid and reliable assessment checklist that can be used to document meaningful, everyday reading, in adults with Down syndrome.

### The significance of reading in the everyday lives of adults with Down syndrome

The impact of how we now use written language in society and therefore the ability to read is particularly pertinent for people with Down syndrome. Firstly, the cognitive and linguistic profile of people with Down syndrome places them at risk for reading difficulties (Abbeduto et al., 2007). Many people with Down syndrome have weak phonological awareness skills (Lemons and Fuchs, 2010) and limited auditory short term memory (Jarrold and Baddeley, 2010; Godfrey and Lee, 2018), both skills that are used during phonic decoding (Cohen et al., 2008). Moreover, even those who have proficient word recognition skills find it difficult to recall details of text (Farrell and Elkins, 1994/1995). Engaging in both tasks simultaneously requires the use of working memory, an ability that is limited for those with Down syndrome (Bird et al., 2001). In addition, people with Down syndrome have language difficulties that are disproportionate to their level of intellectual disability (Abbeduto et al., 2003; Frizelle et al., 2018) and have significant difficulties understanding complex syntax (Frizelle et al., 2018). Consequently, reading comprehension is challenging and even “good readers” show a considerable discrepancy between their ability to recognize words and to understand the text (Boudreau, 2002; Byrne et al., 2002; Nash and Heath, 2011).

The second reason why changes in the use of written language in society are particularly relevant for people with Down syndrome, relates to the fact that an increasing number of people with Down syndrome are engaging independently with local communities to access education, employment and local amenities. This requires a minimum level of reading ability to allow them to complete many of the tasks associated with community living. Increased opportunities for independent living also highlight the need to develop stronger social support networks (Jobling et al., 2000) and to adopt a broader social approach to reading. This is an approach in which reading is viewed as part of living life; the social practices of reading in different contexts are considered: and the meaning that reading has in the lives of those who use it is deemed important (e.g., Papen, 2005; Taylor, 2006). Taking this approach has the potential to allow adults with Down syndrome to experience an increased connection with others and improved access to a range of social and community activities.

### The need for a meaningful literacy approach

Despite the challenges experienced by people with Down syndrome when learning to read, literature shows that people with Down syndrome can and do learn to read to varying degrees (Bochner et al., 2001; Moni and Jobling, 2001; Næss et al., 2012; Reichow et al., 2019). In addition, although early onset cognitive decline is prevalent in individuals with Down syndrome (McCarron et al., 2017), cognitive development continues into adolescence and adulthood (Chapman et al., 1998). Accordingly, there is an increasing acknowledgement in the literature that reading instruction should continue beyond the years of compulsory education. Moreover, it is suggested that the young adult years may be the optimal time to focus on literacy development (Moni and Jobling, 2001) particularly when appropriate teaching and learning strategies are used (Alfassi et al., 2009; Morgan et al., 2013; Browder et al., 2014). Unfortunately, school-based conceptualizations of reading continue to dominate teaching methods, resulting in the marginalization of everyday reading practices (Katims, 2000; Maddox, 2008). Reading instruction needs to move beyond book based activities that only support the development of phonological awareness and phonic decoding skills. A purely functional approach to reading has also been criticized where only sight word instruction is used to teach words that are focused on basic survival (such as STOP or TOILET signs). Authors have highlighted that this makes up only one small part of reading and that a functional focus can often be at the expense of the development of reading for communication, education, participation and pleasure (Cologon, 2012). If programs are to be relevant for adults with Down syndrome they need to combine a functional with a social practices approach to reading (Street, 2003), to note the intention for reading, and to consider how people with Down syndrome construct reading in different contexts (Morgan et al., 2013). Instruction should also include popular culture as well as topics that are of interest and meaningful to people with Down syndrome in their everyday lives (Moni and Jobling, 2008). We refer to this as a *meaningful literacy* approach and use the term in a similar manner to Deagle and Damico (2016).

One approach in which both functional and social practices of reading are considered (*meaningful literacy*) is the Literacy and Technology Hands-On (LATCH-ON) post-school program of instruction for adolescents and adults with Down syndrome (Moni and Jobling, 2000). The program is based on the assumption that those who participate in literacy activities in their communities do so for reasons that are meaningful to them and for desired outcomes. Consequently, the program modules make explicit connections between what participants read and discuss, to events in their family and community lives.

### Searching for appropriate measures of reading in the community

Although findings from the LATCH-ON program clearly indicate that adults with Down syndrome can continue to learn to read (Moni et al., 2018), gains reported have been variable and small for some participants (Moni and Jobling, 2001). In addition, the authors noted difficulties in finding appropriate measures of reading related literacy skills to measure the impact of this type of program. They highlighted that the use of measures such as the Neale Analysis of Reading Ability–Revised (Neale, 1999) or the Woodcock Reading Mastery Tests–Revised (WRMT-R) (Woodcock, 1987), could not adequately capture reading in a socio-cultural context and suggest that a broader range of qualitative measures or observations might be more appropriate. In particular, these measures do not capture change in adults perceptions of themselves as readers and are not meaningful in relation to the everyday lives of people with Down syndrome and the purpose for which they use reading.

Down Syndrome Ireland has been a provider of further education courses for people with Down syndrome in Ireland since 2012, and has provided data to the University of Queensland, Australia, to examine the longitudinal effects of the LATCH-ON program. A recognition that these standardized reading tests were inadequate for the purposes of measuring *meaningful* reading led us to search for a different measure that would allow us to document reading in everyday environments. Unable to find an existing assessment or checklist designed to explore everyday meaningful use of reading skills in this population, we chose to work together with adults with Down syndrome to co-construct an assessment checklist. A valid and reliable checklist could be used as a baseline and outcome measure for post-school reading programs and to guide the content of modules to ensure that programs are meaningful to everyday social practices and reflect participants’ own preferences/choices. This type of checklist may be more sensitive to change in relation to the range of items read pre- and post-intervention programs and could also complement standardized tools that are more focused on more specific skills, such as decoding.

### Psychometric properties of an assessment tool

To use an assessment checklist in clinical practice or for research purposes, it must have evidence of sound psychometric properties (Andersson, 2005; Dockrell and Marshall, 2015). These include the overarching concepts of validity, reliability and responsiveness (Mokkink et al., 2010). Reliability is an indication of whether the measurement tool gives consistent results each time it is used. One indicator of reliability is *internal consistency* which is an index of how far the different items that make up a scale are measuring a common construct or idea. A second reliability measure is referred to as *external reliability* and is a measure of how consistent the scores are between two or more test sessions taken in close proximity (test–retest reliability). In contrast, validity indicates how well a test captures what it sets out to assess. It is measured in a number of ways and includes concepts such *as ecological, face, content,* and *concurrent validity*. *Ecological validity* examines whether the items included in the test are reflective of those in real life settings. *Face validity* refers to whether the test appears to assess the target reading practices in question and includes aspects such as the overall appearance of the test and how it is presented. *Content validity* is the degree to which the content of the test is an adequate reflection of the construct being measured. For example, the content should reflect a wide range of items so that respondents have the opportunity to portray the extent of their skills in a given area. *Concurrent validity* refers to how well the scores on a new measure correspond to those on well established “gold standard” tests for the same children. Note, we do not include all measures of validity in this study as *predictive validity* for example is more appropriate to child populations where significant development is expected regardless of any intervention input. In addition we have not measured *responsiveness* (the ability to detect change over time in the construct being measured) as participants were engaged in different programs over varying time frames and it was not possible to use the tool to measure their skills at baseline.

### Current study

This study was carried out in two stages. In stage one we used an inclusive participatory research design in which adolescents and adults with Down syndrome were partners in the research. This design is collaborative such that (1) the research is undertaken *with* rather than *about* people with Down syndrome (Walmsley, 2004) and (2) their input / opinions and perspectives are integral to the work carried out. The adults with Down syndrome who worked with us in stage 1 are referred to throughout as our *collaborators*. In stage 2 we have taken a more traditional approach and refer to this cohort as *participants* throughout the study.

The following research questions are addressed:

#### Stage 1

- What items should be included in an assessment tool that can be used to profile meaningful reading in adolescents and adults with Down syndrome?
- How should the tool be presented so that (1) it is accessible for people with Down syndrome (2) it has acceptable face and content validity?

#### Stage 2

- To what degree is the Meaningful Reading Measure (MRM) developed reliable?
- Is there a relationship between meaningful reading scores and (1) age, (2) receptive vocabulary, and (3) reading ability as measured by standardized assessments.
- What do adults and adolescents read in their day to day lives?
- How is meaningful reading experienced by adults and adolescents with Down syndrome in relation to what, when, where and why they read; their preferred medium; what is hard about reading; the best thing about reading; and what they would like to be able to do with their reading?

With reference to measuring concurrent validity usual practice is to examine the relationship between the new measure and the “gold standard” currently in use (with the expectation that a relationship will exist) and the investigation is about determining the strength of that relationship. Where a gold standard does not exist, the investigation is one of determining if any relationship exists (hypothesis testing). Given that there is no recognized reference standard for measuring meaningful reading in adults with Down syndrome, our comparisons with standardized tests were therefore considered to be hypothesis testing (rather than measures of concurrent validity). This is in keeping with the Consensus Based Standards for the Selection of Health Status Measurement Instruments (COSMIN) taxonomy^1^ (Mokkink et al., 2010). We did not anticipate a relationship between receptive vocabulary and meaningful reading score. Vocabulary assessments are developed so that the test items increase in difficulty/abstractness as one progresses through the test. In contrast, our meaningful reading checklist is designed to reflect a broad range of items in a real-world context, rather than items that increase in difficulty. Similarly, we did not anticipate a relationship between our standardized reading checklist and meaningful reading score. This hypothesis was based on the fact that our tool was developed to capture changes in the range and number of items that people with Down syndrome may read in a socio-cultural context, a need that is currently not met by standardized reading measures (Moni and Jobling, 2001). Given (1) that reading underpins both our meaningful measure and standardized measures and (2) the relationship reported between vocabulary knowledge and word identification abilities (Wise et al., 2007) one might argue that a relationship is possible, although we did not anticipate relationships would exist. Therefore, we needed to examine this empirically to be sure if our hypotheses were correct.

With respect to potential relationships between meaningful reading score and age, we could have hypothesized a negative association, as younger adults with Down syndrome are less likely to experience cognitive decline than those who are older (McCarron et al., 2017). In addition, they are more likely to have been educated in a mainstream school, and therefore have better reading outcomes (de Graaf et al., 2013). On the contrary we could have anticipated a positive association with age, as older adults have greater life experience and are therefore more likely to be engaged independently with education, employment, or their local community for a longer period, giving them greater exposure to the type of items that would be included in the checklist.

## Methods

### Ethics statement

Ethical approval was granted from the Clinical Therapies Social Research Ethics Committee at University College Cork.

### Stage 1—creation of assessment tool

#### Research partners/collaborators

Forty-six adults with Down syndrome were recruited in to stage 1 of the study. Adults were recruited through three adult education classes, two delivered online and one in-person, all administered through Down syndrome Ireland (DSI). DSI is an organization which offers support and services for people with Down syndrome and their families in Ireland. The final author facilitated study recruitment by liaising with the adult education course teachers. The education courses were offered to all adult members of DSI in 2021, regardless of reading ability. Down syndrome Ireland take a universal design approach to learning and therefore there are no literacy, academic or social pre-requisites to participating in the classes. Online classes required access to the internet and an online platform. The aims of the classes were to build friendships and to develop literacy skills in a very broad sense. All collaborators spoke English as the primary language of the home and based on teacher report, were of mixed cognitive ability. We did not assess the overall cognitive ability of collaborators as we deemed this to be inappropriate in the context of developing a meaningful reading tool with adults and were not using IQ as an inclusion/ exclusion criterion. This is in keeping with Greenspan and Woods (2014) who suggest that arbitrary IQ test scores provide little insight into the relative cognitive strengths and weaknesses of adults with ID. Demographic information for collaborators from each of the classes is given in Table 1.

**Table 1:** Collaborator demographics for checklist creation group *n* = 46.

Class	Age range			Male		Female	
	(Years)	n	%	n	%	n	%
Online adult cohort	18–29	24	52.2	12	26.1	12	26.1
Online teenage cohort	16–18	13	28.3	5	10.9	8	17.4
In-Person cohort	20–55	9	19.5	3	6.5	6	13
Total by gender		46	100	20	43.5	26	56.5

#### Procedure

##### Creating the tool

Collaborators attending each of the three classes were given an easy read information sheet with visual supports explaining the purpose of the study and what would be involved. The information sheet was also read orally by the course teacher who explained that those who wished to be included would complete an optional additional literacy exercise. Collaborators were also invited to ask the researchers, their teachers or parents for further information if needed. This is in line with the Health Service Executive (2022), to maximize a person’s capacity to consent through supported decision making. Collaborators were provided with a written consent form, requiring a tick box response, or could choose to give consent verbally. The consent form was modeled on a previous form which was co-designed with the Down Syndrome National Advisory Council (a committee of people with Down syndrome). The first stage of the literacy exercise was to notice and write down all the things the participants read in a week. The task was outlined on the first teaching day of the week (Monday for the online courses) and a discussion took place in the class or online about potential items that participants might read each day. Results were gathered within 1 week of the initial discussion. Three students were absent when the majority of the class completed the task and they elected to complete it on their return.

**Figure 1:**
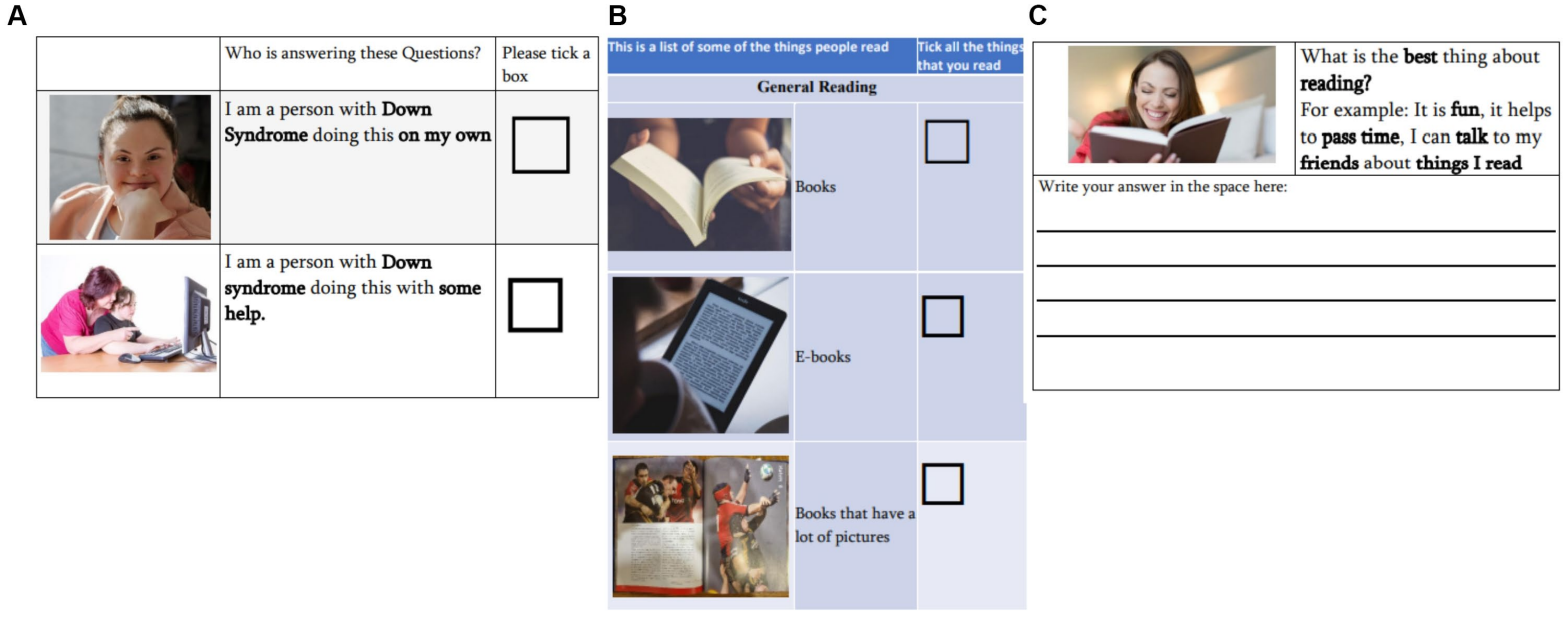
Sample image from version one of the meaningful reading measure.

All anonymized results were shared with the researchers who compiled the lists into one draft checklist and created the first version of the Meaningful Reading Measure (MRM). Guidelines on easy-read materials from Nomura et al. (2010) and the UK Department of Health (2010) were considered in the design of all aspects of the tool. Questions were concise with limited use of abstract language and picture supports were provided to support the meaning of each question. The layout contained wide margins with consistent spacing. Most text was placed inside a defined space in a clear non-serif font and a type-size of 18 pt. or greater. In addition, key words were boldened for emphasis (see Figure 1).

Version 1 of the MRM contained three sections. Section 1 focused on demographic information, including age; whether collaborators were completing the checklist independently or with support; and educational information, such as number of years spent in mainstream/special education and any additional courses completed (Figure 1A). Section 2 included a list of all items that collaborators stated they read. Items that were very similar were conflated (e.g., text message, what’s app message) resulting in 42 unique items. Each item was depicted visually with supported text and a tick box which would allow collaborators to indicate which items they typically read. Each ticked item would be counted as 1 which when summed would result in a total meaningful reading score. Space was also allowed for participants to add items which were not covered in the checklist. Figure 1B shows an example of how the items were depicted. Section 3 included 7 additional questions on collaborators’ reading, to provide a context for collaborators’ responses and to inform future courses where meaningful reading is an area of focus. The first was a closed question about the medium through which collaborators read and why, with a number of options *Do you most like to read—a paper book, an eBook, on a tablet or computer, listening to audiobooks, I do not like to read.* The remaining questions were open-ended: *why do you read?; where do you read?; when do you read?; what is the best thing about reading?; what would you like to be able to do with your reading?; and what is hard about your reading?* (Figure 1C).

##### Establishing face and content validity

**Figure 2:**
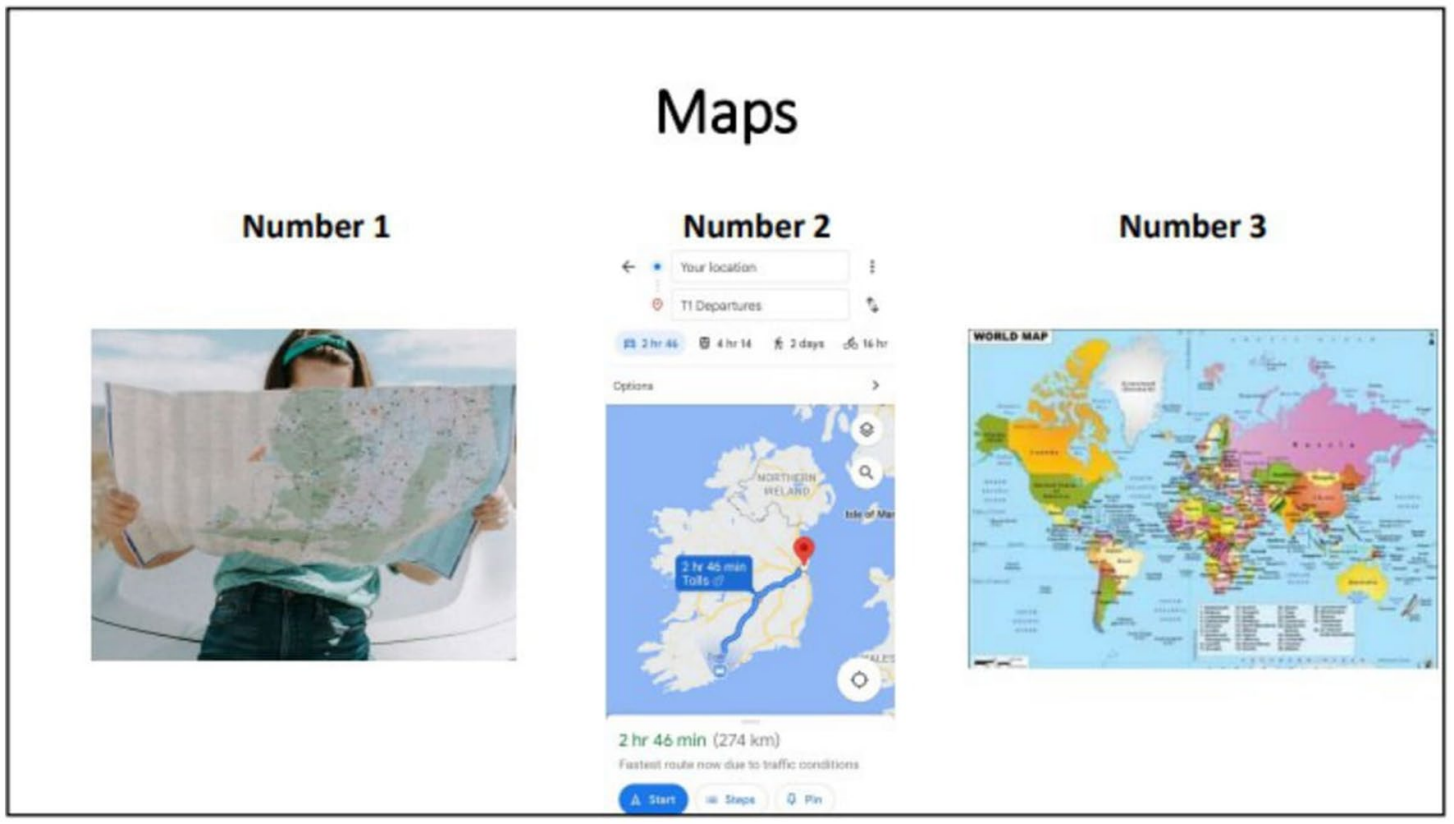
Sample from image validation exercise.

To establish face and content validity the MRM was then shared in electronic and hard copy formats with the 10 collaborators who were completing their adult education course in person (a sub-group of the original 46). Those who attended the online adult education classes were not included at this stage as they had completed the classes and moved on to other things. The process of obtaining feedback was iterative. The third author sent the checklist to the class teacher and asked him to get the participants’ views on the MRM. An in-class discussion took place and the teacher reported back verbally to say that the collaborators liked all aspects of the MRM. We reflected on this process and realized that a request for general feedback was not an adequate method to generate feedback on specific elements of the tool. Consequently, we revised our methodology. Regarding the main section of the MRM (the list of items), we identified 15 image supports from the checklist which we deemed to be either (a) visually unclear, (b) potentially problematic for someone with visual difficulties, or (c) a poor representation of the concept the image was intended to represent. Additional images were sourced for each of these 15 items, to ascertain which image depicted the concept most clearly. These images formed the basis of a PowerPoint presentation that was given to the 10 participants with Down syndrome through the online conferencing platform Zoom. The online conference call was conducted by 3 members of the research team and in addition to the collaborators with Down syndrome, was attended by the class teacher and two teaching assistants. The class teacher acted as a facilitator for any collaborator who had difficulty understanding any aspect of the process. The PowerPoint presentation was given by the second author and consisted of 15 slides each containing 3 different images to represent the same concept. Figure 2 shows an example with reference to the item *Maps*. Collaborators were then asked which picture depicted each item most accurately/clearly. Responses were tallied and the image for which the majority of the group voted was chosen to represent each item in the checklist.

On the basis of collaborators’ responses a second version of the MRM was created to include the validated image choices and a reorganization of the items such that they were presented in the following categories—general reading; personal reading; reading when out and about; and reading a screen/reading for fun. The MRM was sent to the same 10 collaborators so that they could complete the second iteration of the checklist as an in-class exercise, facilitated by their class teacher. Prior to completing the MRM collaborators were asked to appraise the measure with reference to (1) ease of completion, (2) items included, and (3) layout/overall appearance. Immediately following completion, a class discussion took place where collaborators discussed what they liked and did not like about the measure, as well as any further modifications that should be made. The in-class exercise was followed by an online focus group in which the researchers asked for final feedback on the measure. Again the class teacher acted as a facilitator, rephrasing questions when required and asking specific collaborators for their input. The focus group was audio recorded and transcribed by the second author (SOD). SOD then coded responses with one of three codes. The code *affirmation* was used to indicate a positive comment about an element of the measure; *alteration* was used to indicate a recommendation to change or remove an element; and *additional information* was used to reflect comments pertaining to the length of the survey, ease of completion and general feedback. Suggested modifications were taken into account and the final version of the MRM for teenagers and adults with Down syndrome was created.

### Results

#### Stage 1

##### Version 1

**Figure 3:**
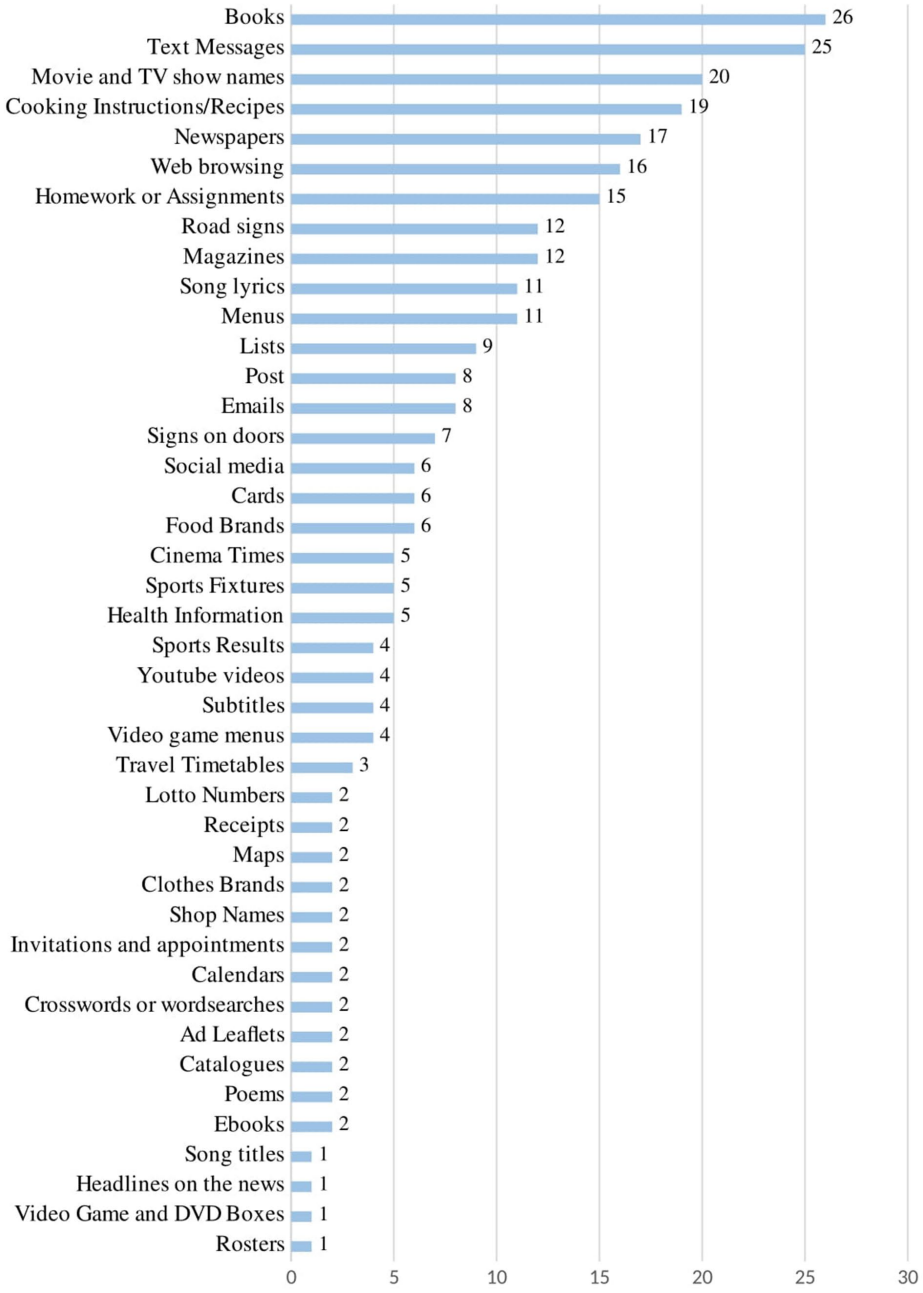
Frequency of reading items listed by collaborators.

In addressing our first research question we aimed to establish what items should be included in an assessment measure that could be used to profile functional reading in teenagers and adults with Down syndrome and how that measure should be presented (i.e., the ecological, face and content validity of the measure). Responses from 46 collaborators indicated that 42 items should be included in the first version of the checklist. A frequency analysis of the items listed was conducted and is shown in Figure 3. Following a review by the research team an additional 4 items were added—books with picture supports; museum displays; contracts; and board games. This resulted in a total of 46 items in the first version of the MRM.

##### Face and ecological and content validity

Results from the image validation process indicated that only two of the previously used images were chosen by adults with Down syndrome to remain in the MRM, namely those that represented *emails* and *board games*. The group voted on an alternative image for the remaining 13 representations. For each image there was a majority vote. The images presented through PowerPoint along with the voting tally are shown in Supplementary Figure S1.

Results from the focus group affirmed the following features of the measure—the use of visual images and supports (such as bolded text) with all questions rather than confining these supports to the checklist part of the measure; the addition of examples for sections 1 and 3 (the demographic and opinion questions); the use of a blue background color to enhance the checklist portion of the tool; and the use of sub-headings to reflect different categories of reading within the checklist. In relation to alterations, collaborators suggested increasing the font size throughout the measure; increasing the space provided for collaborator responses; the inclusion of lines to assist with response presentation; and the addition of two DSI further education course options, which had been completed by most of the collaborators. Finally, “additional information” that was noted in the focus group related to guidance on how the tool might be completed in the future. The majority of collaborators (70%) stated that their preference would be to complete the measure using pen and paper rather than using a computer. Seventy percent also stated that they would prefer to complete the measure as part of a class exercise with the assistance of their teacher, rather than independently at home or with the assistance of a family member. All recommended alterations were made to the measure before progressing to stage 2 of the study.

### Stage 2

Stage 2 included two parts. The first examined test–retest reliability and associations with standardized tests and the second examined the lived experiences of adults with Down syndrome.

#### Participants

In order to reduce the burden on any one group, different cohorts were recruited into the second stage of the study. To establish external reliability 23 adults with DS were recruited into the test re-test component of stage 2. These were a convenience group of adults who were studying on adult education courses run or supported by DSI and all spoke English as the primary language of the home. Participants were recruited through the third and final authors who contacted the course teachers, informed them of the study, and asked them to impart the information to those attending their course. As before, the course teacher shared the study information sheet and consent form (developed in an easy read format) with the course participants and read the information aloud, to ensure informed consent. Of the 23 that gave initial consent, 3 were absent on the day of MRM completion and 1 opted not to complete the MRM the second time. Therefore 19 adults with Down syndrome participated in this aspect of the study.

Twenty-five adults with DS were recruited into the next part of stage 2—exploring potential relationships between the MRM, age and standardized test results. These participants were a cohort for whom standardized literacy and vocabulary assessments had been recently completed as baseline and outcome measures for their literacy program. The participants were either attending an adult literacy course administered by DSI or were in the process of enrolling in one. The recruitment process was similar to that outlined above. Those in the process of enrolling in an adult literacy course were contacted directly by the 3rd author and invited to take part. The 3rd author read the information sheet and consent form to the potential participants and was available to ask any questions. Initially all of those invited elected to take part in this aspect of the research, however 1 changed her mind, 1 was absent on the day the checklists were completed and PPVT scores were not available for 2 participants. Consequently, the final number included was 23 for both reading measures and 21 for the vocabulary measure. There was an overlap of 12 participants between the test re-test group and those who completed the standardized assessments.

Thirty three participants were recruited to complete the final part of stage 2 (i.e., what adults and adolescents read and how they experience reading in their day to day lives). These included all of those who completed the test retest; those who did the standardized assessments; and 2 additional participants who were absent on the day the MRM retest was completed. For those who completed it twice, we report the MRM results from the first timepoint. Demographic information for each cohorts is given in Table 2.

**Table 2:** Participant demographics for stage 2.

	Test–retest group (*n* = 19)				Standardized assessment group (*n* = 24)			
	*M*	*SD*	Median	Range	*M*	*SD*	Median	Range
Age	30	8.67	29	21–53	30.58	8.81	28.5	19–53
Sex (M:F)	10:9				15:9			
PVVT (*n* = 22)					92.32	33.74	80.5	47–257
BURT (*n* = 24)					29.13	5.97	26.5	0–96

#### Measures

##### Peabody picture vocabulary test

The Peabody Picture Vocabulary test-4th Edition (PPVT-4)(Dunn and Dunn, 2007) was used to measure receptive vocabulary. This is a norm-referenced standardized test which can be used from 2½ to 90 years. It has test–retest reliability coefficients of 0.92 and 0.96 and split half reliability of 0.94 and 0.95. Standard scores are based on a typically developing population and are therefore not suitable for people with intellectual disability. Consequently, raw scores were used in the current study. Participants are shown four color pictures on each page. The test administrator says a word that describes one of the pictures and the participant is asked to indicate which one of the four pictures is being described.

##### Burt reading test

The Burt Reading Recognition test (Gilmore et al., 1981) was used to measure participants’ ability to read single words. The test consists of a list of 110 real words arranged in groups of 10, presented in decreasing size and increasing order of difficulty. The test was developed for use with typically developing children up to 12 years old. It is recommended to stop testing following 10 consecutive errors. The total number of words read correctly yields a raw score and this can be converted into a reading age. We did not deem reading age to be an appropriate metric for adults with intellectual disability and have used raw scores in this study.

#### Procedure

Participants completed the MRM in hard copy in the room in which they attended their adult literacy course or in the center in which their course would be delivered. Those already attending a course completed it in one sitting at the same time as their peers. Those enrolled in a course came to the education center individually and completed it while the 3rd author was present. Teacher or researcher support was available in both contexts and was dependent on individual preferences. Some adults chose to sit one to one with a teacher or teaching assistant, while others completed the task in small groups (no greater than 4 adults to one teacher providing support). In the group context teachers introduced the task to the whole group and some adults initially expressed doubts about their ability to read. In response, the teachers explained that the MRM was not just about reading books but was developed to document all types of reading that might occur in the participants’ daily lives. Support given also involved reminding participants to look at each item; to turn the page; and to ask if they needed a break. Teachers also gave positive feedback and encouraged participants to get to the end of the task. Those who completed the measure twice did so within 1 week. Alpha numeric codes were added to participant response sheets so that meaningful reading scores could be cross-referenced (a) time 1 with time 2 and (b) with vocabulary and reading test results. All data were anonymized within DSI before sharing with the first and second authors.

#### Data analysis

**Figure 4:**
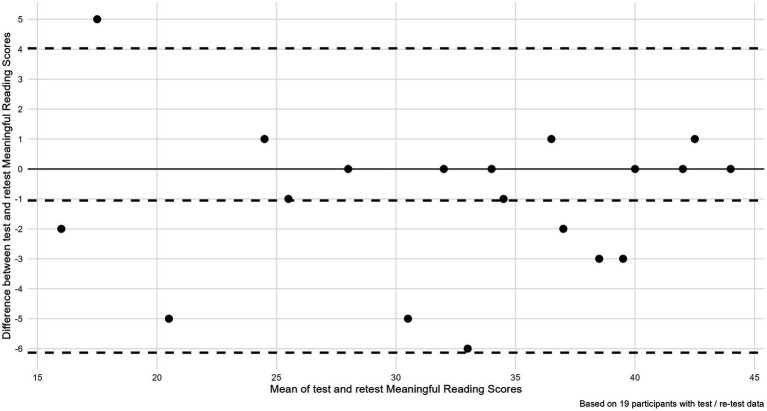
Bland–Altman plot for agreement in test and retest meaningful reading scores.

Internal consistency was measured using Cronbach’s alpha. Test–retest (external) reliability was initially examined using a Spearman correlation coefficient for paired samples. However, because the correlation measures only the strength of the relationship between the two variables, but not the agreement, we also completed a Bland–Altman analysis (Bland and Altman, 1995) and calculated a Coefficient of Repeatability. In a Bland–Altman plot (Figure 4) the difference between the test–retest scores is plotted against the mean of the scores for each time point. This method allows us to calculate the mean difference between the two times the assessment was completed (the “bias”) and 95% limits of agreement of the mean difference (1.96 SD).

Spearman correlations were carried out to examine if there were relationships between total meaningful reading score (based on the first completion of the measure) and results from standardized vocabulary and literacy assessments. Participants’ raw scores were used for the standardized vocabulary and literacy assessments as these assessments have not been standardized on populations with intellectual disability, to minimize potential floor effects, and to avoid obscuring individual differences by utilizing standard scores (e.g., Kover and Atwood, 2013). Quantitative data on demographics, and meaningful reading profiles were analyzed descriptively. Finally, a qualitative directed content analysis (Hsieh and Shannon, 2005) was completed on the data collected from section 3 of the measure (i.e., responses to the open-ended questions that would allow us to profile in more detail participants’ meaningful reading practices). All responses were transferred into NVivo in preparation for analysis. Steps taken were those outlined by Hsieh and Shannon (2005). Data were read repeatedly by the third author to achieve immersion and a sense of the whole data set. Data were then read word by word, and words from the text that captured key predetermined concepts (e.g., where, when, and why do you read) were highlighted to derive codes. Codes were then organized into categories based on how different codes were related. Incidence of codes representing each category were noted under each of the six question headings. To increase trustworthiness all data and coding was discussed with and reviewed by the first author.

## Results

Our first research question in stage 2, was to establish to what degree the MRM was reliable (i.e., what is the internal and external reliability of the measure?). Of the 33 participants 31 had a complete data set. Cronbach’s alpha (Kuder–Richardson formula-20, used for dichotomous scores) showed an internal consistency value of 0.93, indicating a homogenous test. We examined test–retest reliability of scores based on our sample of 19 participants. The estimated paired sample Spearman correlation was high at 0.95 (95% CI 0.88–0.98), while the ICC estimated with a linear mixed effects model was 0.96. We also evaluated agreement in test re-test scores using a Bland–Altman analysis (Bland and Altman, 1995). The mean difference was −1.05 and the 95% limits of agreement were −6.13 to 4.03. Visual inspection of the Bland–Altman plot did not reveal any concerning patterns or trends (see Figure 4). Finally (based on a within subjects SD of 1.93), the Coefficient of Repeatability showed that the difference between 2 observations for the same person is estimated at <5.35 points for 95% of observed pairs.

Our second research question addressed whether there was a relationship between meaningful reading and (1) age, (2) receptive vocabulary, and (3) reading ability as measured by a standardized vocabulary and reading assessment, respectively. Given that the MRM data were not normally distributed, Spearman’s rank correlations were completed. Results indicated no significant relationships between meaningful reading score and age (*r* = 0.12, *p* = 0.57); receptive vocabulary (*r* = 0.01, *p* = 0.96); or reading ability as measured by the Burt word recognition test (*r* = −0.05, *p* = 0.79).

**Figure 5:**
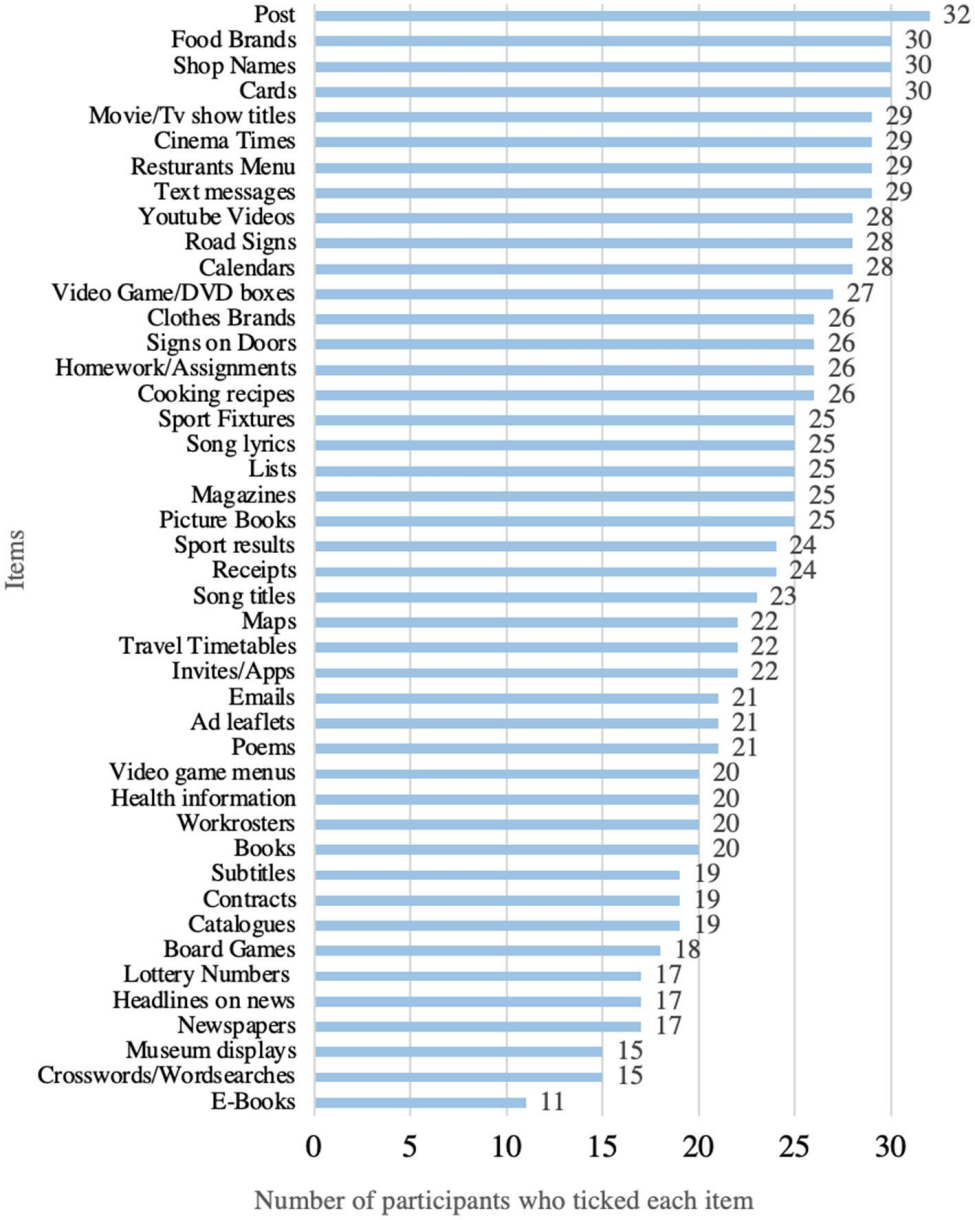
Frequency with which each item was read from stage 2 participants (*n* = 31). This graph does not present data on 2 items (social media and web browsing) which were accidently excluded from the checklist presented to these participants.

Our third research question asked what adults and adolescents read in their day to day lives. Results for the mean number of total items read (Meaningful Reading Score) are given in Table 3 and the frequency with which each item was ticked is shown in Figure 3. The lowest score was 12 and the highest was 44, indicating that everyone who participated read a minimum of 12 items on the checklist in their everyday lives. As shown in Figure 5 almost all participants (≥91%) ticked that they read *post*, (e.g., *letters/ postcards, items received by mail*), *food brands*, *shop names*, and *cards*. Approximately 50% of participants stated that they read *lottery numbers, headlines on the news* and *newspapers*. *E-Books* were the least common item read (33% of participants).

**Table 3:** Checklist results for complete cohort.

	Average reading checklist score (*n* = 31)			
	*M*	*SD*	Median	Range
Reading score	31.06	9.49	34	12–44
Age	28.94	8.41	26	19–53
Sex (M:F)	21:12			

**Figure 6:**
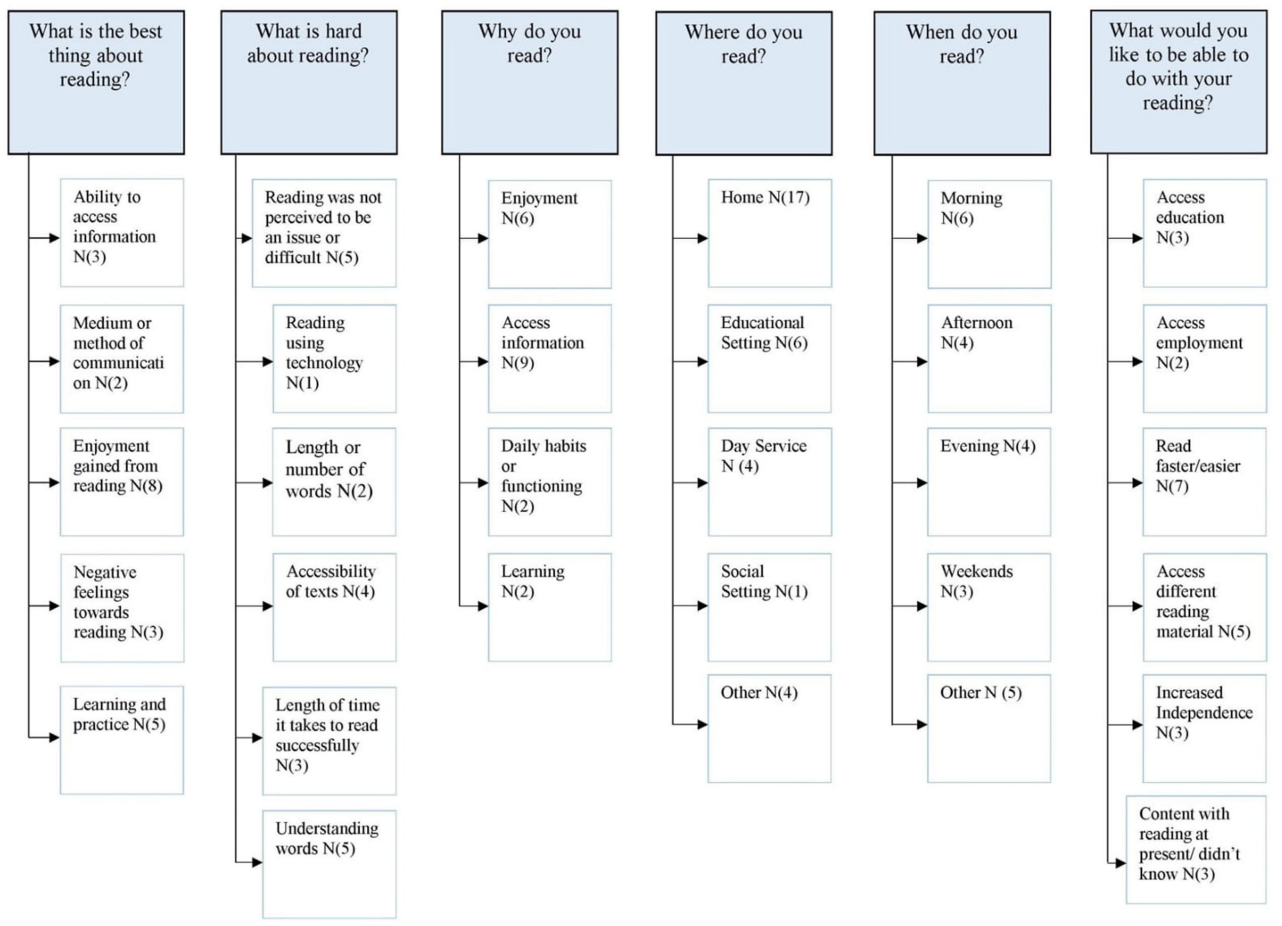
Content analysis of questions in section 3 of the assessment checklist. N = number of participants in each category.

Our final research question addressed how meaningful reading is experienced by adults with Down syndrome in relation to when, where and why they read; their preferred medium; what is hard about reading; the best thing about reading; and what they would like to be able to do with their reading? Our qualitative analysis of these questions (asked in the final section of the meaningful reading checklist) is summarized in Figure 6.

The first question was a closed question (using a tick-box format) and asked participants about the medium in which they preferred to read and why (*Which do you most like to read? Why?*). Most participants chose to read on a tablet or computer (*n* = 12); some preferred a paper book (*n* = 7); others stated that they do not like to read (*n* = 7); some did not have any preference (*n* = 4); one participant preferred listening to audio books and no-one reported reading eBooks or using a kindle. The remaining questions were all open-ended, the first of which asked participants what they considered to be the best thing about reading. The most common response was that reading was pleasurable/enjoyable. One participant described the immersive nature of reading “I can read books; it is fun and is [like] a TV in my head” while another enjoyed it as a relaxing activity “just relaxing my brain.” Some participants (*n* = 3) described how reading enabled them to access information or find out more about their interests, “[it] gives me information.” Others (*n* = 5) felt that reading was helpful for learning and a way to practice getting better at other activities “Helps me to focus and learn big, long words, helps me with my phone.” A couple of participants (*n* = 2) stated how reading helped them communicate with others, and others (*n* = 3) expressed negative feelings toward reading, “I was better years ago.” In the next question participants were asked what they felt was hard about reading. The two most common responses to this question were that reading is not difficult (*n* = 5), “I do not think it’s hard” and that understanding words is difficult (*n* = 5), “words I do not understand….it takes me *for* months to read one story.” Participants also had issues with small print and with reading long words. When asked why they read, most said that they read to access information (*n* = 9), “I read to find out SOAP spoilers” or “To learn new things… to find out information.” Others stated that they read for enjoyment (*n* = 6); because it was a daily habit (*n* = 2); and to aid their learning (*n* = 2), “to learn new ways to spell, syllables.” In the next question participants were asked where they read. Most stated that they read at home (*n* = 17); others responded that they read in an educational setting (*n* = 6), others read in their day service (*n* = 4), and one participant reported reading in a social setting. Other respondents said that they read only when they had to, “Only when I have to…, texts on my phone when they come in.” Participants were then asked when they read. Responses were varied and included during the morning, afternoon, evening, and weekends. Five participants did not specify a particular time of the day and stated, “I do not have time to read” and “Quiet time with my mum…. midday prayers.” In the final question, participants were asked what they would like to be able to do with their reading. The most common response was that participants would like to be able to read faster and more easily (*n* = 7); some wanted to access other material (*n* = 5) “I would like to read on my phone”; others wanted to use reading to help them achieve greater levels of independence (*n* = 3) “Put money in my [my bank account] my boss put in” and “[Read] on my phone and computer and without help”; some participants did not have any goals for their reading (*n* = 3); and a few would like their reading to help then access employment (*n* = 2) and further education (*n* = 3). More detailed quotes under each category are given in Supplementary Tables S1, S2.

## Discussion

### Stage 1

In the current study we initially aimed to establish (1) what items should be included in an assessment measure that could be used to profile meaningful reading in adolescents and adults with Down syndrome and (2) optimal presentation to ensure the measure has acceptable face and content validity and is accessible for people with Down syndrome. Ultimately, this type of assessment tool could be used as an outcome measure for post-school reading programs and could serve to complement standardized tools as it would potentially be more sensitive to change, regarding the range of items read pre- and post-intervention. We argue that an increase in range of items read represents one aspect of reading growth, which in the longer term could result in the ability to read a greater number of words. Importantly, an increase in range of items read has the potential to increase confidence and motivation when engaging with written material. In addition, it can facilitate individuals with Down syndrome to participate in richer social practices in their community, much of which can be achieved through engagement with a broader range of written material (e.g., through social media), without increasing the difficulty level of words presented. Additionally, the MRM could be used as a measure of cognitive decline. In practice, in early stages of dementia, parents often report a reduction in their son’s/daughter’s ability to engage with meaningful reading tasks that are part of their daily lives, for example finding the television channel they want. One would expect that these changes are more likely to be reflected in a meaningful reading measure than in standardized reading assessments. However, further work would need to be carried out longitudinally to examine sensitivity of the task to change over time.

Stage 1 of our study was participatory in that adolescents and adults with Down syndrome were active collaborators/research partners throughout the process of constructing the tool. Ninety-one percent of the items included in the final version of the tool were generated by 46 individuals with Down syndrome, with the remaining 9% (4 items) added by the research team. Following the generation of the items, our method of collaborative practice in relation to face and content validity was problematic, in that when asked in a general way, all collaborators with Down syndrome stated that they liked all aspects of the measure. This was a reminder to the research team that we needed to be much more specific in our approach. We then focused on how each item was visually depicted and identified 15 image supports that we deemed to be visually unclear; potentially problematic for someone with visual difficulties; or that could be represented in multiple ways. Alternative images were sourced for each of these items and collaborators were asked to vote on their preferred representation, by raising their hand. Using this method gave our collaborators a specific area to critique and allowed all members of the group to actively contribute, independent of reading or spoken language ability. Consequently, 13 of the 15 images were changed from how they were originally depicted, indicating that the images we had originally chosen were not optimal for our collaborators with Down syndrome. This is keeping with that reported by Sutherland and Isherwood (2016) who found that although photographs/images are helpful, they can often be confusing and do not always convey the correct message. Following this process, our collaborators indicated that they really enjoyed the exercise and that they had never been asked to contribute to research like this before. The realization that the images would be changed based on their opinions appeared to increase their confidence and level of involvement in the final feedback stage. Final feedback was informed by a class discussion and an online focus group, in which our collaborators were asked to focus on specific aspects of the measure, such as how easy it was to complete, the items included and the layout/overall appearance. In this stage of the process collaborators were increasingly vocal, were very forthcoming about specific aspects of the measure that they liked and gave several suggestions regarding how the presentation of the measure could be improved. Suggestions, which included increasing the font size and space provided for participant responses, were reflective of the guidelines put forward by Nomura et al. (2010) who highlight the range of layout interventions required to make a document easier to read and comprehend. It was interesting that most collaborators stated that their preference would be to complete the tool using pen and paper (rather than on a computer). This is perhaps unsurprising, given some of the ongoing computer usability challenges evinced by individuals with Down syndrome (e.g., password usability) (Kumin et al., 2012), as well as variability regarding formal computer training among our collaborators. In addition, they stated that they would like to do it as part of a class exercise with the assistance of an adult educator, rather than at home. However, both responses may just reflect our collaborators’ experiences in this study and if given the opportunity to complete the tool online or at home, it is possible that they may equally embrace this experience.

### Stage 2

#### Reliability

In the second stage of the study, we aimed to establish if the measure was reliable. Our findings clearly indicate both internal and external reliability. Internal reliability is shown by a Cronbach’s alpha of 0.93, which is established in the literature as an indicator of strong reliability (Taber, 2018). In addition, although there were some individual differences in our test–retest data, a Spearman correlation of 0.95 indicates a strong relationship between the first and second time the tool was completed (external reliability). This was further supported by the agreement levels shown in our Bland–Altman analysis (mean difference of −1.05), an ICC of 0.96 and a small coefficient of repeatability (<5.35).

#### Relationship with other variables

In our next research question, we asked if there was a relationship between meaningful reading scores and (1) age and (2) standardized measures of receptive vocabulary and reading ability. Our data clearly shows no relationship between meaningful reading score and these other variables. In relation to age, as we stated at the outset, we could have argued for a positive or negative association. Negative, in the context of younger adults with Down syndrome being (1) less likely to experience cognitive decline (McCarron et al., 2017) and (2) less likely to have been educated in a special school and therefore more likely to have increased reading skills (de Graaf et al., 2013). Positive, because of the greater life experiences of older adults which gives them greater exposure to meaningful text reflected in the items in the checklist. It is possible that all these factors were at play (with the effect of one factor negating the effect of another) and therefore no clear relationship emerged. It is also noteworthy that we did not account for cognitive ability in our analysis, a factor that is not independent of our findings.

A lack of relationship between our measure and the standardized vocabulary assessment (PPVT-4) is not surprising and was in keeping with our hypothesis. Most standardized vocabulary checklists are based on a developmental trajectory and tend to reflect vocabulary that might be relevant to primary and post-primary education rather than to socio-cultural experiences in the community. Even tests that have been normed on typical adult populations tend not to include areas relevant to community living, such as popular culture. Vocabulary in typical adults is measured as a construct that increases in difficulty (i.e., levels of abstractness with a focus on more academic language) rather than vocabulary quantity, reflecting a broader range of topics. In contrast, growth in receptive vocabulary development in adolescents and adults with Down syndrome is more likely to be driven by individual, educational, environmental, social, and cultural experiences. Conversations with some of the participants in the current study suggested continuing vocabulary development in areas such as sport, local and national politics and Covid-19, none of which would be reflected in a standardized measure such as the PPVT-4.

It was also unsurprising that there was no relationship between meaningful reading score and The Burt Reading Recognition Test (our standardized reading measure). While some of our participants were unable to read even the first line of the Burt (which consists of the words *to, is, up, he, at*), all participants indicated that they read some of the items on the checklist (a minimum of 12 items). In terms of sight word recognition, words such as t*o, is, up, he, at* tend not to represent the most pertinent information in a sentence and therefore become less relevant to reading key points of information that facilitate functioning in everyday life. The ability to read other words in the Burt (such as *projecting, explorer, domineer*) is reflective of decoding skills without any context and is far removed from reading for a specific purpose where the context provides significant support and the act of reading is underpinned by a different motivation. We did not expect an association between meaningful reading and this standardized word recognition test. As has been previously highlighted in the literature (see Moni and Jobling, 2001), standardized measures like this do not adequately capture reading in a socio-cultural context. Particularly, they do not capture change in how adults perceive themselves as readers. Some participants in the current study described themselves as non-readers at the outset. However, when encouraged to look at the MRM they found the experience to be empowering, and it allowed them to notice the ways that they read the written word in everyday life. Consequently, having completed the measure they began to identify as readers.

#### Meaningful reading in everyday lives

Our final two research questions addressed what meaningful reading is for adolescents and adults with Down syndrome. Although there was considerable individual variation, all participants indicated that they read some of the items in the MRM (a minimum of 12 and a maximum of 44). Items read, reflected a broad range of reading practices from *restaurant menus, sport fixtures, travel timetables* and *poems* to *lottery numbers* and *wordsearches*. The span of items is indicative of the importance of examining and targeting a range of reading practices within our educational contexts (Street, 2001). In addition, our dataset can guide educationalists designing and developing post-school literacy modules to ensure that programs reflect the full extent of everyday reading practices for this cohort. With respect to where and when individuals with Down syndrome read, our data shows *at home* to be the most popular response and reinforces the idea that reading is an activity that is not confined to educational settings for people with Down syndrome. No clear pattern emerged in relation to when individuals with Down syndrome read, indicating that meaningful reading is integral to people’s lives at different times throughout the day. Regarding the most important thing about reading, the majority of responses referred to the fact that reading was an enjoyable activity and that it is something that aids learning. As noted by Williams (2005), pleasure and enjoyment have not been a priority in post-school literacy courses, which tend instead to focus on employment based skills. Despite the fact that it may enhance quality of life, literacy for pleasure and recreation has been neglected (Ashman and Suttie, 1995). Our data reinforces the view that this needs to change. In addition, responses indicating that reading is something our participants did to aid their learning and that they read to access information, demonstrates an ongoing interest in lifelong learning for people with Down syndrome. In keeping with the United Nations’ Convention on the Rights of Persons with Disabilities (UNCRPD) it is important that adolescents and adults with Down syndrome are given equal opportunities to access relevant education and training (art. 24) throughout their lives (United Nations, 2006).

### Limitations and future steps

There are some limitations to the current study which we note here. Firstly, our information sheets were developed by the research team. Although the team had significant experience working with people with Down syndrome, given the collaborative nature of the work (stage 1) it would have been preferable that our collaborators were integral to this process. Secondly, given that 13 of the 15 images presented to our collaborators (depicting each item) were exchanged, one could argue that we should have asked for feedback on the images that represented all items in the checklist. We did not do this as we believed that some images (such as *receipt*) were easy to depict in a universal manner and we wanted to reduce the burden of the task for our collaborators with Down syndrome. The images we chose for validation were those we deemed to be potentially problematic and we were therefore not surprised that such a large proportion of these were revised. Thirdly, two items were accidentally omitted from the final checklist (i.e., social media and web browsing). Given the number of times they were generated by our collaborators in stage 1, we expect that they would have featured strongly in the items most often read by our stage 2 participants. If it is the case that these items are frequently read by adolescents and adults with Down syndrome, it would support the need to include popular culture in post-school programs, which would serve to build social capital and develop common frames of reference between those with Down syndrome and their “typical” peers (Davies and Dickinson, 2004). Lastly, given that we did not expect a relationship between standardized reading scores and our meaningful reading measure, it would have been preferable to have also included a measure (such as reading engagement) for which a relationship may have been more likely. That said, these measures exist for the general population only and therefore may not be appropriate or may require significant adaptation for people with Down syndrome. As a potential additional validation, we did pilot asking parents to complete the checklist (without picture supports) but it became clear that unless reading books, parents did not view their sons/daughters as readers. It may have been a fruitful exercise to offer “training” to parents in the purpose of the measure and to ask them to observe and document their son or daughter’s reading over a specified period.

Regarding future steps, while our sample is representative of a range of adolescents/adults with Down syndrome in Ireland (many of whom attend some form of further education), the sample is relatively small and the measure would now need to be used to profile a much larger group nationally and internationally. To strengthen measurement of change over time it may also be useful to develop a supplemental sheet for family members which could capture frequency data (regarding how often the individual with Down syndrome is engaging with meaningful reading tasks) as well as qualitative information on changes in behavior. This would include the ability to engage in new reading tasks as well as tasks that individuals were previously able to do. Finally, it would be useful to complete longitudinal work to investigate the sensitivity of the MRM in measuring change in reading behavior over longer periods of time.

## Conclusion

This study reports on the development of a measure of broad-based reading-related literacy skills in collaboration with a group of adolescents and adults with Down syndrome. Our preliminary data presented here shows that the measure is reliable as well as having strong ecological and content validity. As an outcome measure, the MRM can serve to complement existing standardized tools and can be used to measure change regarding the range of items read pre- and post-reading intervention programs (although further work is required to establish the measure’s sensitivity to change over time). The MRM can also guide post-school reading program content to ensure that it is meaningful to the everyday social practices of people with Down syndrome. Lastly, by framing reading as a meaningful daily activity, the MRM can capture growth in these adults’ perceptions of themselves as legitimate readers, reflecting an increased confidence and motivation to read. Consequently, it can help educationalists and others in society to recognize adults with Down syndrome as valued literate members of the community in which they live.

## Data availability statement

The original contributions presented in the study are included in the article/Supplementary material, further inquiries can be directed to the corresponding author.

## Ethics statement

The studies involving human participants were reviewed and approved by the School of Clinical Therapies Social Research Ethics Committee, University College Cork. Written informed consent to participate in this study was provided by the participants’ themselves who were given documentation in advance of the study to discuss with their parent/guardian if they wished to.

## Author contributions

PF conceptualized the study and wrote the manuscript. SO’D engaged with collaborators to generate the tool and participants to test it (test–retest reliability) and completed some of the standardized vocabulary and reading tests. MJ completed some of the test–retest reliability work and the qualitative data analysis. LM completed some of the standardized tests, facilitated recruitment and assisted with focus group. NH conceptualized the study, facilitated recruitment, completed some test–retest reliability work, assisted with focus group and provided comments on manuscript. All authors contributed to the article and approved the submitted version.

## Conflict of interest

The authors declare that the research was conducted in the absence of any commercial or financial relationships that could be construed as a potential conflict of interest.

## Publisher’s note

All claims expressed in this article are solely those of the authors and do not necessarily represent those of their affiliated organizations, or those of the publisher, the editors and the reviewers. Any product that may be evaluated in this article, or claim that may be made by its manufacturer, is not guaranteed or endorsed by the publisher.

## Footnotes

^1^ http://www.cosmin.nl
