# Supplementary material for: The co-construction of a reading assessment measure with adults with Down syndrome: a meaningful literacy approach
Source: Front Psychol. 2023 Jul 20;14:1173300. doi: 10.3389/fpsyg.2023.1173300 (PMC10399224; doi:10.3389/fpsyg.2023.1173300)
Supplement: Supplementary file 1 [file Data_Sheet_1.docx]

**Table S1 - *Qualitative analysis***

| **Question Prompt** | **Theme** | **Quotes** |
| --- | --- | --- |
| **What’s the best thing about reading?** | Ability to access information | *N(3)*  *“Gives me information”*  *“I like books about sport”* |
|  |  |  |
|  | Medium of communication | *N(2)*  *“I can talk to my friends on text”*  *“[I can] talk about [favourite show]”* |
|  | Enjoyment gained from reading | *N(8)*  *“I can read books, it is fun and is [like] a TV in my head”*  *“Getting into the classic adventure”* |
|  | Negative feelings towards reading | *N(3)*  *“I don’t like it”*  *“I was better years ago”* |
|  | Learning and practice | *N(5)*  *“Helps me to focus and learn big long words, [reading] helps me with my phone”*  *“It is good for your head”*  *“Learning”* |
| **What is hard about reading?** | Reading was not perceived to be an issue or difficult | *N(5)*  *“I don’t think it’s hard”*  *“It’s so simple”* |
|  | Reading using technology | *N(1)*  *“on my iPhone”* |
|  | Length or amount of words | *N(2)*  *“ I find it harder if the words are too long”*  *“Too many words, big words”* |
|  | Accessibility of texts | *N(4)*  *“Words are too small”*  *“Words are too small, I don’t understand some words”* |
|  | Length of time it takes to read successfully | *N(3)*  *“I find it hard to [pronounce] a word I don’t understand”*  *“It takes too long”* |
|  | Understanding words | *N(5)*  *“There are words I don't understand”*  *“words I don’t understand.. it takes me for months to read one story”* |
| **Why do you read?** | Enjoyment | *N(6)*  *“I love it”*  *“I read because it inspires me to write a story of my own”* |
|  | Access information | *N(9)*  *“To learn new things… to find out information”*  *“I read to find out SOAP spoilers”*  *“To read about what they are doing in big time - soccer or acting fixtures”* |
|  | Daily habits or functioning | *N(2)*  *“It’s good to read”*  *“To write things down”* |
|  | Learning | *N(2)*  *“To learn new things”*  *“To learn new ways to spell, syllables”* |
| **Where do you read?** | Home | *N(17)* |
|  | Educational Setting | *N(6)* |
|  | Day Service | *N(4)* |
|  | Social Settings | *N(1)* |
|  | Other | *N(4)*  *“Only when I have to, texts on my phone when they come in”*  *“I read at work”* |
| **When do you read?** | Morning | *N (6)* |
|  | Afternoon | *N (4)* |
|  | Evening | *N (4)*  *“At night time when I go to bed”*  *“Before bed”* |
|  | Weekends | *N (3)* |
|  | Other | *N(5)*  *“I don’t have time to read”*  *“Quiet time with my mum, midday prayers”* |
| **What would you like to be able to do with your reading?** | Access Education | \| *N(3) –*    *“College”* \| \| --- \| \| *“I want to do a creative writing course”* \| |
|  | Access employment | *N(2) “To get a job”* |
|  | Read faster/ easier | *N(7)*  *“I would like to read faster and read more words also on my phone*  *“To read without help, to read more words”* |
|  | Access different reading material | *N(5)*  *“I would like to read on my phone””*  *“Reading more newspapers”* |
|  | Increased Independence | *N(3)*  *“Put money in my [my bank account] my boss put in”*  *“[Read] on my phone and computer and without help”*  *“Read without help"* |
|  | Content with reading at present/ didn’t know | \| *N(3)*  *“I don’t know”* \| \| --- \| \| *“It is hard to think”* \| \| *“No - I don’t want to be better”* \| |

**Table S2 *Qualitative drop down options and responses***

| **Which do you most like to read?** | | **Why?** |
| --- | --- | --- |
| A paper book | *N(7)* | *“I just do, it keeps me up to date”*  *“It gives me a break from screens”*  *“Because it’s easier with glasses”*  *“Good in my hands”*  *“Can get It in the library”*  *“to learn new things”* |
| An E-book like a kindle | *N(0)* |  |
| On a tablet or computer | *N(12)* | *“I text my friends”*  *“So I can read emails, I use the iPad at home”*  *“It keeps me up to date”*  *“Because I love using technology”*  *“Because I have them at home, online, college, cinema times , phone, texts*  *“ I can make words smaller”*  *“ I can make the words big”*  *“great to have”*  *“movie stories”*  *“I work, I read spreadsheets at work”*  *“Can look up more information about areas”* |
| Listening to audiobooks | *N(1)* | *“I love listening”* |
| I don’t like to read | *N(7)* |  |
| Others who wrote instead:  “[I like them] all the same”  “Good for information” | *N(4)* | *“ I like my family tree book”* |
